# The effects of Tai Chi practice on plantar tactile sensation and ankle proprioception in older adults with peripheral neuropathy

**DOI:** 10.3389/fspor.2026.1780973

**Published:** 2026-06-17

**Authors:** Qiongqiu Zhao, Qi Wang, Ziyin Liu, Qingqing Song, Xiaoxue Zhu, Qipeng Song

**Affiliations:** 1 Graduate School, Shandong Sport University, Jinan, China; 2 Biomechanics Laboratory College of Human Movement Science, Beijing Sport University, Beijing, China; 3 College of Sports and Health, Shandong Sport University, Jinan, China; 4 College of Physical Education and Sport, Beijing Normal University, Beijing, China; 5 School of Exercise and Health, Shanghai University of Sport, Shanghai, China

**Keywords:** brisk walking, older people, peripheral neuropathy, sensation, Tai Chi quan

## Abstract

**Purpose:**

Peripheral neuropathy (PN) leads to decrease plantar tactile sensation and ankle proprioception in older adults, thereby elevating the risk of falls. While exercise has proven effective in mitigating these deficits, comparative studies on the effects of different exercise modalities remain scarce. This study aimed to investigate the effects of Tai Chi (TC) and Brisk Walking (BW) on plantar tactile sensation and ankle proprioception in older adults with PN.

**Methods:**

A total of 38 participants who met the inclusion and exclusion criteria were randomly assigned to either the TC practice (12 female, 6 male; age: 71.5 ± 5.3 years; height: 160.3 ± 8.4 cm; weight: 65.7 ± 11.7 kg) or the BW practice (11 female, 7 male; age: 71.6 ± 5.6 years; height: 158.7 ± 6.7 cm; weight: 64.9 ± 8.5 kg). Each practice regimen lasted 8 weeks and consisted of three 60-minute sessions per week, with an exercise intensity maintained at 55%–65% of the estimated maximum heart rate (HRmax). Plantar tactile sensation threshold and ankle proprioception threshold were assessed at baseline (week_0_) and immediately after the intervention (week_9_) (a lower detection threshold indicates higher tactile sensation in that area). Statistical analyses were performed using two-way repeated-measures ANOVA for normally distributed data or generalized estimating equations (GEE) for non-normally distributed data.

**Results:**

Significant interaction effects were observed for plantar tactile sensation threshold at both fifth metatarsal head (*p* = 0.007, OR = 0.111, 95%CI: 0.023−0.544) and the heel (*p* = 0.002, OR = 0.059, 95%CI: 0.010–0.355). Simple effect analysis revealed that TC practitioners showed greater reductions in plantar tactile sensation threshold at fifth metatarsal head and the heel compared to BW practitioners. Additionally, a significant interaction effect of ankle proprioception threshold was found during plantarflexion (*p* < 0.002, *η*^2^_p_ = 0.355), with TC practitioners showing a more significant reduction than BW. A time effect for ankle proprioception threshold during dorsiflexion (*p* < 0.001, *η*^2^_p_ = 0.611) across both practices.

**Conclusion:**

TC demonstrated superior effectiveness compared to BW in decrease plantar tactile sensation threshold and ankle proprioception threshold in elderly individuals with PN.

## 1 Introduction

Peripheral neuropathy (PN) is a prevalent neurological disorder, particularly among older adults, with an estimated prevalence of 3.3% to 8% in those aged 55 and older (1), attributed to physiological degeneration and increased incidence of conditions like diabetes and cancer (2). PN manifests in various forms, with diabetic neuropathy being the most common, followed by idiopathic, toxic, and inflammatory neuropathies (3). These conditions are all characterized by damage to the axon and/or myelin sheath, which leads to deficits in sensory and motor systems, particularly in the lower extremity, and increases the risk of falls due to sensory loss in the feet (4).

Older adults, in general, rely heavily on plantar tactile sensation and proprioception (about 70%) to maintain postural stability (5). However, for those with PN, this reliance is compromised. PN leads to weakened plantar tactile sensation, which provides essential information about foot contact pressure and directly impacts balance (6). Additionally, proprioception, particularly at the ankle joint, also declines in PN (7), proprioception is the perception of limb movement and spatial orientation generated by body stimulation (8). Impairments in these sensory functions significantly increase fall risk (4), which is 15 times higher compared to age-matched individuals with intact sensation (9). Falls have been proven to be the leading cause of death among elderly individuals worldwide (4).

While the effect of Tai Chi (TC) on improving plantar tactile sensation and ankle proprioception in the elderly has been confirmed in previous studies—demonstrating its effectiveness in improving age-related somatosensation in individuals over the age of 60, with improvements observed in both tactile sensation and ankle kinesthesia (10). The same author cohort has proven that TC was beneficial for both elderly individuals with sensory loss and those without, enhancing cutaneous sensitivity and ankle proprioception (11); notably, in the sensory loss group, improvements were observed in more areas. Although sensory loss is an early symptom of PN and TC has proven effective for it (11), PN damages the sensory system by impairing axons and myelin, slowing nerve conduction velocity, and compromising sensory conduction (12). Furthermore, this type of neural damage is difficult to cure (13). Given these pathological changes, it remains unclear whether the beneficial effects of TC observed in healthy or age-related sensory decline populations can be extrapolated to older adults with established PN.

Therefore, this study aims to investigate the effects of TC practice on plantar tactile sensation and ankle proprioception in older adults with peripheral neuropathy. It is hypothesized that 1. TC practice and regular exercise (like brisk walking, BW) will improve plantar tactile sensation and ankle proprioception among older adults with PN, and 2. the practicing of TC will lead to greater improvement in plantar tactile sensation and ankle proprioception than BW.

## 2 Materials and methods

### 2.1 Sample size estimate

The required sample size for this study was calculated using G*Power 3.1 software (G∗ Power Software Inc., Kiel, Germany), setting the alpha level at 0.05 (*α* = 0.05) and statistical power at 0.95 (*β* = 0.95). Based on prior research that compared the ankle proprioception before and after TC or BW interventions in two groups of elderly individuals (TC_before_ = 1.94 ± 0.40°, TC_afte*r*_ = 1.15 ± 0.20°; BW_before_ = 2.12 ± 0.47°, BW_afte*r*_ = 1.63 ± 0.53°), with an effect size (*η*^2^_p_) of 0.092 (10), the minimum total sample size was calculated to be 24 participants. To account for participant adherence and potential dropouts, a minimum of 30 participants was deemed necessary.

### 2.2 Participants

A cohort of 43 older adults, aged 60 years and older, was recruited through the distribution of flyers and the delivery of presentations within the local community. The diagnosis of PN was established through a dual approach: initial clinical assessment by a neurologist, followed by confirmation via a plantar pressure threshold detection test to ensure consistency, given the variability in diagnostic criteria among physicians (14); this test, involving three random trials at each of five plantar sites with standardized procedures (15), scored pressure sensation responses (“yes”) where two or more correct responses indicated complete sensation (scoring “1”) and two or more incorrect responses indicated abnormal sensation (scoring “0”), with cumulative scores per site ranging from 0 to 5 (16), and was validated as a conservative method for PN detection due to its lower false positive rate compared to the gold standard electrodiagnostic test (17).

Participants with PN were included if they met the following criteria: (1) a plantar pressure test score of ≤ 3 (16), (2) a physician-confirmed diagnosis of PN, and (3) no regular physical exercise in the past two years, defined as at least 3 months of consistent exercise, three times per week, with each session lasting a minimum of 30 min. Exclusion criteria encompassed foot ulcers, any other movement disorders that could impact physical performance, or uncontrolled cardiovascular, respiratory, or metabolic conditions.

The protocol for this study was approved by the Ethics Review Committee of Shandong Sport University (2025054) and conformed to the principles of the Declaration of Helsinki. All participants provided written informed consent before enrollment.

### 2.3 Tai Chi (TC) intervention

The TC intervention consisted of 8 weeks of training, with 3 sessions per week (60 min/session). During Weeks 1–4, each session included a 5-minute warm-up, 20 min of learning 2 new movements (totaling 24 movements by Week 4), a 10-minute rest, 20 min of reviewing previously learned movements, and a 5-minute cool-down. In Weeks 5–8, sessions transitioned to 40 min of continuous TC practice, integrating all 24 movements, with a 5-minute warm-up and 5-minute cool-down. Intensity was self-regulated, emphasizing smooth, controlled movements without strain. The intensity of TC requires a heart rate of 55%–65% of the estimated maximum heart rate (HRmax), HRmax was monitored using a heart rate chest strap (18).

### 2.4 Brisk walking (BW) intervention

The BW intervention also spanned 8 weeks, with 3 sessions per week (60 min/session). Weeks 1–4 focused on adaptation, with 20 min of slow walking (40%–50% HRmax), a 10-minute rest, and 20 min of BW. Weeks 5–8 progressed to two 20-minute BW segments (55%–65% HRmax), separated by a 5-minute rest.

### 2.5 Plantar tactile sensation threshold test

**Figure 1:**
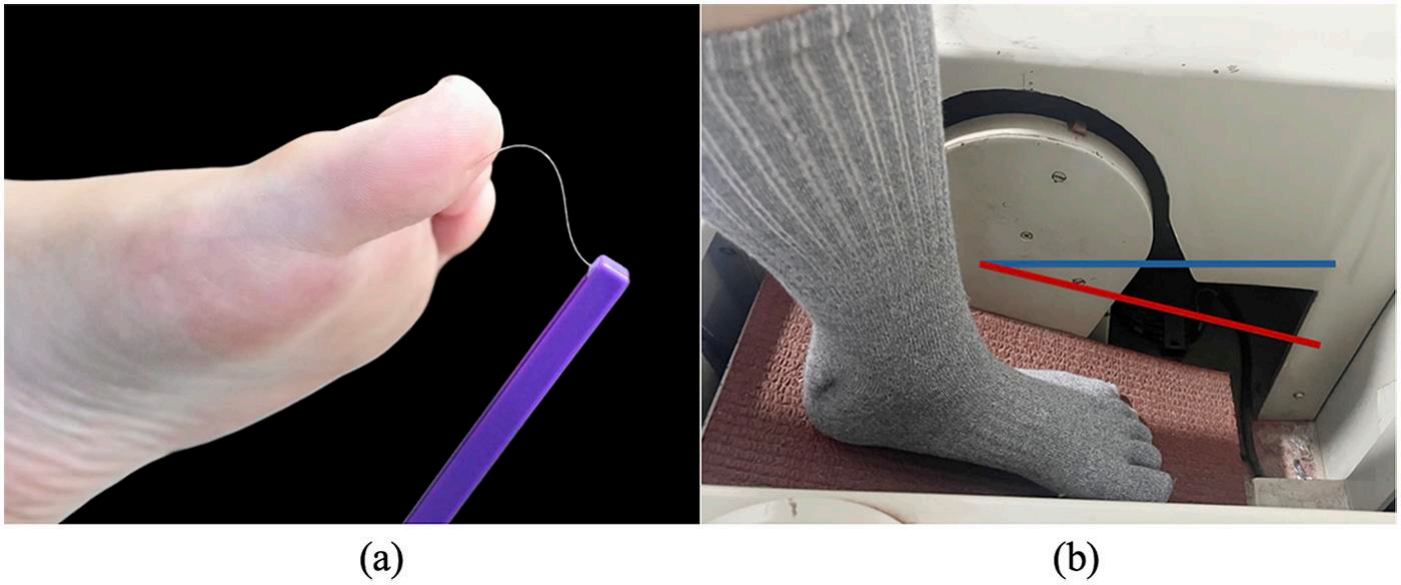
Illustration of plantar tactile sensation and proprioception tests. **(a)** The plantar tactile sensation was assessed using a monofilament test, and **(b)** proprioceptive sensitivity was evaluated with a specialized proprioception testing device. In **(a)**, the monofilament was applied perpendicularly to the skin at the first metatarsal head, bending into a 90˚ C-shape upon contact to ensure standardized pressure. In **(b)**, the proprioception test device displayed a reference horizontal line (blue) and a dynamic line (red) indicating the angular displacement of a rotating pedal. Participants reported the onset of pedal rotation, and proprioceptive sensitivity was quantified as the angular difference (in degrees) between the reference line and the reported rotation threshold.

Plantar tactile sensation threshold was assessed using the Semmes-Weinstein Monofilament (North Coast Medical, Inc., Morgan Hill, CA, USA) (Figure 1a), demonstrating high test-retest reliability (ICC = 0.83–0.86) (19). Testing targeted five sites on the dominant foot (defined as the kicking foot): hallux, first metatarsal head (MH1), fifth metatarsal head (MH5), arch, and heel (20). The monofilament sizes used in this study were 2.83, 3.61, 4.31, 4.56, 5.07, and 6.65, corresponding to forces of 0.07 g, 0.40 g, 2.00 g, 4.00 g, 10.00 g, and 300.00 g, respectively, when bent into a 90˚ C-shape against the plantar skin (15). Each site was tested five times in randomized order, The sensitivity threshold for each site was determined by the thinnest monoﬁlament size the participant could feel (21), a lower threshold indicates better tactile sensation. Randomized null stimuli (no contact) were included to control for anticipation bias.

### 2.6 Ankle proprioception threshold test

Ankle proprioception thresholds were assessed using the AP-11 proprioception test device (Jinan Sunny Intelligent Technology) (Figure 1b), which demonstrated good test-retest reliability (ICC = 0.74–0.94) (22). The device measured the minimum detectable angular motion during ankle dorsiflexion/plantarflexion via a rotating platform (0.4˚/s angular velocity) controlled by a participant-activated hand switch, with displacement recorded by an electronic goniometer. Participants were seated with the knee and hip flexed at 90˚, leg perpendicular to the platform, and ∼50% of lower extremity weight supported by the platform; a thigh cuff suspension system minimized plantar tactile sensory input (23). To control for extraneous stimuli, participants wore headphones (white noise) and kept their eyes closed, pressing the switch upon detecting motion and identifying its direction. The motor initiated rotation after a random 2–10 s delay, with ≥ 5 trials per direction conducted to reduce variability; proprioception sensitivity was defined as the mean angular displacement threshold across trials (24), a lower threshold indicates better proprioceptive sensation.

### 2.7 Statistics

All statistical analyses were conducted using SPSS 27.0 (IBM Corp., Armonk, NY, USA), with the significance level set at *p* < 0.05. Primary analyses were performed following the intention-to-treat (ITT) principle, ensuring that all randomized participants were included in their originally assigned groups, thereby preserving the integrity of randomization. To assess baseline comparability between the TC and BW groups, and to inform the choice of primary statistical model, continuous variables were first tested for normality using the Shapiro–Wilk test. For normally distributed variables, independent samples t-tests were used for baseline comparison; for non-normally distributed variables or ordinal data, the Mann–Whitney U test was employed.

For normally distributed outcomes, a two-way repeated measures ANOVA was used with Group (TC vs. BW) and Time (week0 vs. week_9_) as factors. *Post-hoc* tests employed Bonferroni correction. If no interaction effect was observed (*p* ≥ 0.05), further analysis focused on the main effects of Time and Group. Statistical significance for *post hoc* comparisons was set at *p* < 0.05, with effect sizes reported as partial eta squared (*η*^2^_p_) for ANOVA results (*η*^2^_p_ = 0.01–0.06 (small), *η*^2^_p_ = 0.06–0.14 (moderate), *η*^2^_p_ ≥ 0.14 (large)) and Cohen's *d* for *post hoc* pairwise comparisons (*d* < 0.2 (minimal), *d* = 0.21–0.5 (small), *d* = 0.5–0.8 (moderate), *d* > 0.8 (large).

For non-normally distributed data, Generalized Estimating Equations (GEE) were employed to evaluate the effects of Group, Time, and their interaction. If a significant Group*Time interaction was identified, simple effect analyses were conducted to interpret the interaction by examining the time effect within each group, with Bonferroni correction applied to the two within-group comparisons (TC and BW) within each site, setting the significance threshold at *α* = 0.025 (0.05/2). For GEE models, effect sizes are presented as odds ratios (OR) with 95% confidence intervals. In this study, for the key simple effect of Time (post-intervention vs. baseline), an OR below 1 indicates functional improvement (since lower scores reflect better function), with smaller ORs (closer to 0) reflecting greater improvement.

## 3 Results

**Figure 2:**
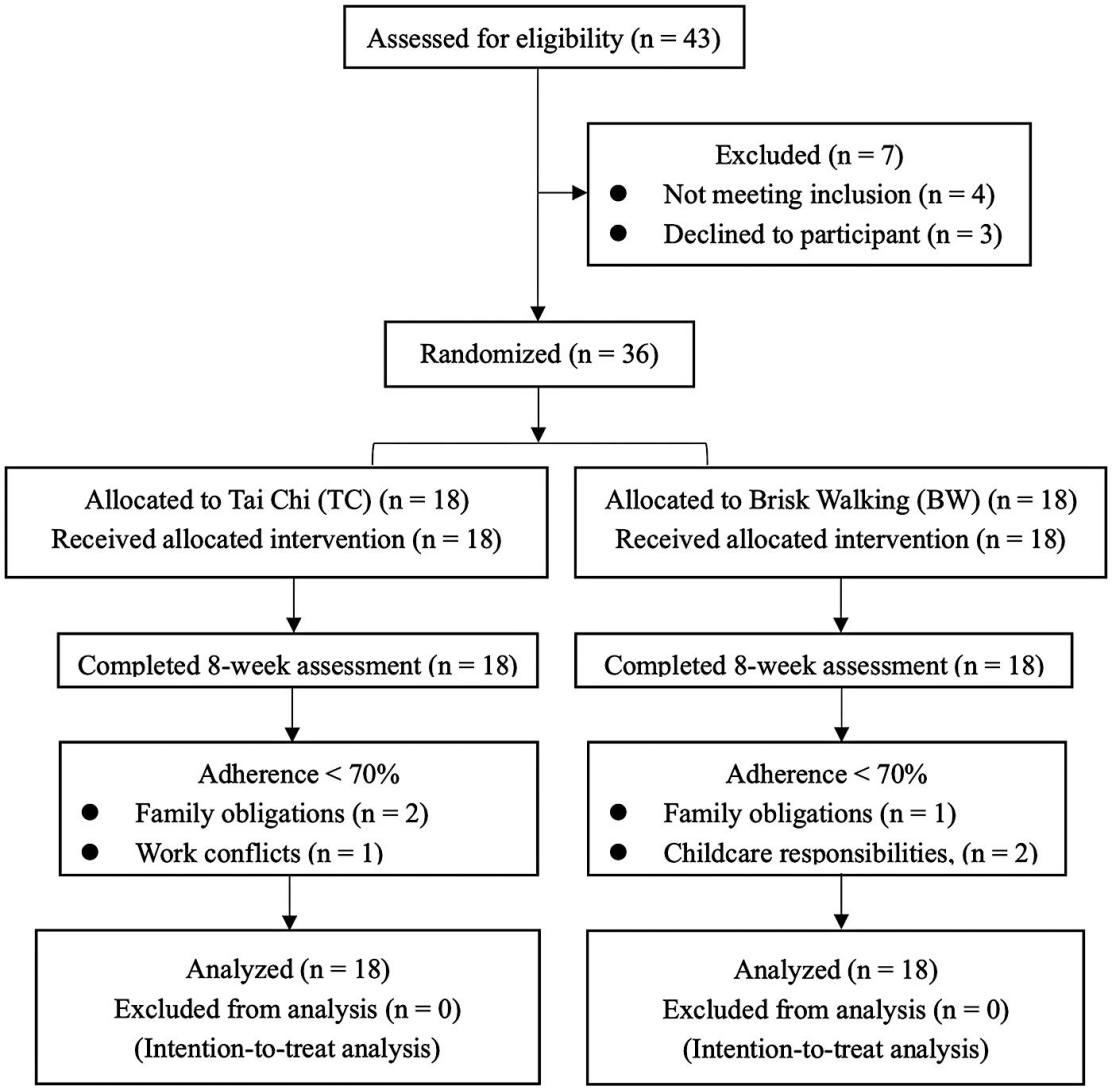
Participant flow chart. A total of 43 participants were assessed for eligibility; 4 were excluded for not meeting inclusion criteria, and 3 declined to practice. The remaining 36 were randomized into TC (*n* = 18) and BW (*n* = 18) practice group. During the 8-week intervention, 6 participants (3 per group) had an adherence rate below 70% due to personal reasons (family obligations, *n* = 3; work conflicts, *n* = 1; childcare responsibilities, *n* = 2). No participants withdrew from the study. An ITT analysis was employed to preserve the integrity of randomization.18 participants completed TC, and 18 completed BW.

A total of 43 participants were assessed for eligibility, of whom 4 were excluded for not meeting inclusion criteria (e.g., insufficient peripheral neuropathy severity) and 3 declined participations. 36 eligible participants were randomized (1:1) into Tai Chi (TC, *n* = 18) and Brisk Walking (BW, *n* = 18) groups. All 36 randomized participants completed the 8-week intervention and assessments. Six participants (3 per group) had an adherence rate below 70% due to personal reasons (family obligations, *n* = 3; work conflicts, *n* = 1; childcare responsibilities, *n* = 2). No participants withdrew from the study (Figure 2). 18 participants completed TC (12 female, 6 male; age: 71.5 ± 5.3 years; height: 160.3 ± 8.4 cm; weight: 65.7 ± 11.7 kg) and 18 completed BW (11 female, 7 male; age: 71.6 ± 5.6 years; height: 158.7 ± 6.7 cm; weight: 64.9 ± 8.5 kg), with no statistically significant between-group differences in baseline characteristics (*p* > 0.05).

As shown in Table 1, a significant interaction effects on plantar tactile sensation thresholds were observed at the MH5 (*p* = 0.007, OR = 0.111, 95%CI: 0.023–0.544) and the heel (*p* = 0.002, OR = 0.059, 95%CI: 0.010–0.355), indicating significantly improved sensory (OR < 1). Simple effect analyses revealed that plantar tactile sensation threshold significantly decreased from week_0_ to week_9_ in both the TC (*p* < 0.001, OR = 0.009, 95% CI: 0.002–0.041) and the BW (*p* < .001, OR = 0.083, 95% CI: 0.023–0.304) practitioners at MH5. Since a lower threshold indicates better sensation, these results demonstrate that both interventions improved plantar tactile sensation at MH5, with TC yielding a superior effect. At the heel, TC practitioners exhibited a significant decrease at the heel from week_0_ to week_9_ (*p* < 0.001, OR = 0.044, 95% CI: 0.008–0.247), while the BW showed no significant change. This indicates that TC practice led to a significant improvement in plantar tactile sensation at the heel.

**Table 1:** Result of generalized estimating equations for plantar tactile sensation threshold.

Plantar sites	Interaction effect: *p*, OR 95% CI	Time main effect: *p*, OR 95% CI	Simple effect analysis
Hallux	*p* = 0.422, 0.705 0.299–1.657	*p* = 0.798, 0.925 0.508–1.683	/
MH1	*p* = 0.192, 2.622 0.617–11.149	*p* = 0.011, 0.213 0.065–0.703	/
MH5	*p* = 0.031, 0.180 0.038–0.860	*p* < 0.001, 0.076 0.019–0.306	TC: *p* < 0.001, 0.014 0.003–0.059 BW: *p* < 0.001, 0.76 0.019–0.306
Arch	*p* = 0.800, 0.898 0.391–2.063	*p* = 0.377, 0.875 0.652–1.176	/
Heel	*p* = 0.032, 0.167 0.033–0.854	*p* = 0.583, 0.763 0.290–2.006	TC: *p* < 0.001, 0.044 0.008–0.247 BW: *p* = 0.583, 0.763 0.290–2.006

OR, odds ratio; CI, confidence interval; MH1, first metatarsal head; MH5, fifth metatarsal head. Time reference: week 0 (baseline); Group reference: brisk walking (BW) group. An OR < 1 indicates a lower probability of a higher sensory score post-intervention relative to baseline. Since a lower score represents better sensory function, OR < 1 signifies functional improvement. The strength of the effect increases as the OR approaches 0. Bonferroni correction was applied to within-group comparisons within each site (*α* = 0.025); all reported significant within-group effects remained significant after correction.

**Figure 3:**
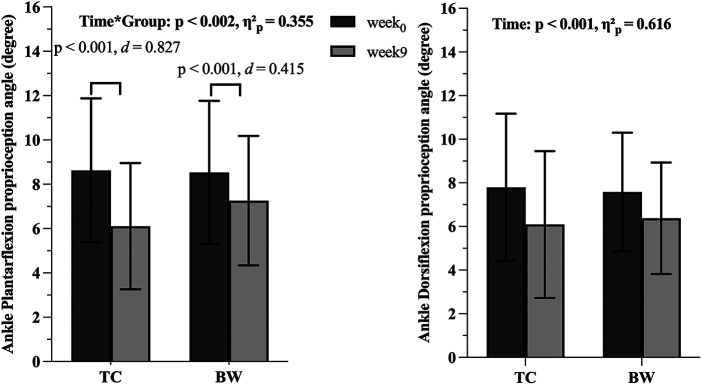
Ankle proprioception threshold before and after interventions. Error bars represent ± 1 standard deviation. *P*-values indicate significant main effects of Time, Group, and Group*Time interaction. Partial eta-squared and Cohen's *d* were used to measure effect size.

As shown in Figure 3, a significant interaction effect on ankle proprioception threshold was observed during plantarflexion (*p* < 0.002, *η*^2^_p_ = 0.355). *Post hoc* tests indicated that ankle proprioception thresholds during plantarflexion were significantly reduced from week 0 to week 9 in both the TC (*p* < 0.001, *d* = 0.827) and the BW practitioners (*p* < 0.001, *d* = 0.415). Since a lower threshold indicates better ankle proprioception, this result demonstrates that TC practice led to a significantly greater in ankle proprioception improvement than BW. A significant time effect was detected for the ankle proprioception threshold during dorsiflexion (*p* < 0.001, *η*^2^_p_ = 0.616), suggesting an overall reduction in the threshold across both practices. This indicates that ankle proprioception during dorsiflexion was significantly improved following the interventions.

## 4 Discussion

The results indicated that both TC and BW exercises improved plantar tactile sensation and ankle proprioception in elderly individuals with PN. TC was more effective than BW at enhancing MH5 and heel position on the sole, as well as ankle proprioception during plantarflexion. These findings supported hypotheses #1 and #2. The findings of this study carry both theoretical and practical implications. Theoretically, our results suggest that for individuals with PN, TC—an exercise emphasizing slow, mindful postural adjustments—may be more effective than purely rhythmic BW in stimulating and remodeling residual sensory pathways. This provides new evidence for understanding the rehabilitation mechanisms of task-specific exercise interventions in people with PN. Practically, this study may offer a basis for designing fall-prevention programs for older adults with PN, indicating that TC should be considered a preferred recommendation over BW. Clinicians and rehabilitation therapists could incorporate standardized TC routines into the regular management of this population, especially for those with severe impairments in plantar tactile sensation and proprioception.

### 4.1 Tai Chi (TC) and brisk walking (BW) practices improve plantar tactile sensation

After 8 weeks of TC practice, there was a significant improvement in plantar tactile sensation at the MH5, arch, and heel. Our findings were consistent with a previous study, which demonstrated that 16 weeks of TC practice enhanced plantar tactile sensation in older adults with sensory loss (11). The slow and precise movements of TC deliver sustained and diverse sensorimotor signals to the brain, providing the patterned, repeated stimulation that may induce Long-Term Potentiation (LTP), leading to synaptic remodeling and consequently improving plantar tactile sensation. A synaptic strengthening mechanism that is critical for neural plasticity (25), has been observed in the hippocampal dentate gyrus following TC practice (26). Specifically, TC promotes LTP at synapses between low-threshold mechanoreceptors (LTMRs) and spinal dorsal horn neurons, thereby improving plantar sensory processing and functional plasticity (27). Given that PN involves synaptic weakening, TC-induced LTP may counteract this decline and enhance sensory recovery. Moreover, as a gentle and slow exercise, TC stimulates both Aβ-LTMRs and C-LTMRs (28), which are responsible for light and slow touch sensation. After 8 weeks of BW practice, there was a significant improvement in plantar tactile sensation at the MH5 and arch. The literature on BW-specific effects remains sparse. Kluding and colleagues reported enhanced plantar vibration perception after combined walking and resistance training (29). The underlying reason may be that Aβ-LTMRs are also stimulated during BW (28), similar to TC.

### 4.2 Tai Chi (TC) had superior efficacy in enhancing plantar tactile sensation

After practice, TC practitioners exhibited significantly greater improvements in plantar tactile sensation at the MH5 and the heel compared to BW. Although no prior studies have directly compared these interventions for plantar tactile sensory outcomes, we assume that their distinct movement patterns account for TC's superiority. TC's lateral sidestepping specifically targets the foot's high-density sensory receptors along the lateral border (30), providing more effective stimulation at MH5 than BW's linear motion (31). Additionally, TC's intentional heel rotations (32) activate the sparsely distributed receptors in the heel (30), which BW minimally engages, potentially leading to a reduction in sensory threshold at the heel only after TC practice. This mechanistic divergence highlights how TC's multidirectional, deliberate movements optimize plantar sensory input more comprehensively than BW's habitual gait patterns. Furthermore, TC stimulates more LTMRs than BW; and although BW can induce LTP (25), its effect may not be as pronounced as that of TC. This is because the induction of structural LTP, which underlies synapse formation in specific motor cortical regions, is primarily associated with learning novel and complex motor skills (33). Since BW is a conventional exercise, it does not fulfill this requirement.

### 4.3 Tai Chi (TC) and brisk walking (BW) practices improve ankle proprioception

The ankle proprioception was significant improved during plantarflexion and dorsiflexion after 8-weeks TC practice. A previous study Zhang et al. supported us by demonstrating that the TC practitioners showed significantly greater improvement in proprioception during both dorsiflexion and plantarflexion directions in older adults without PN (24). Three reasons account for how TC enhances ankle proprioception.

First, TC practice may trigger a series of physiological responses through muscular mechanical contraction, potentially upregulating Brain-Derived Neurotrophic Factor (BDNF) synthesis, which could ultimately contribute to improved proprioception. Specifically, the mechanical and electrical events of muscle contraction during TC lead to a rise in intracellular calcium concentration (34). This calcium signal activates key pathways, such as cAMP Response Element-Binding Protein and Nuclear Factor of Activated T-cells, triggering the transcription and synthesis of BDNF (35). The subsequent increase in BDNF, a well-established factor in neural development, synaptic plasticity, and LTP (36–39), facilitates the regeneration of axons in peripheral sensory nerves, ultimately leading to enhanced proprioceptive function.

Second, TC's mechanic stimulation may promote axonal regeneration and myelination by Schwann cells, thereby enhancing proprioceptive function. PN is defined by pathological processes that result in damage to axons, their myelin sheaths, or both (4). However, peripheral nerves possess remarkable plasticity, enabling axonal regeneration (40, 41) and remyelination (42) that facilitate functional recovery in affected regions, this regenerative capacity is largely dependent on and supported by Schwann cells (43), the myelin-forming glial cells of the peripheral nervous system, which clear degenerated debris (44, 45) and subsequently assist in axonal regeneration and remyelination (46). TC may enhance proprioception through a defined molecular pathway: by stimulating mechanoreceptors, it boosts neural electrical activity, prompting active neurons to release the signaling molecule Neuregulin-1 (47). The binding of Neuregulin-1 to Erythroblastic Oncogene B receptors on Schwann cell surfaces then initiates powerful intracellular signaling cascades (48). These cascades directly instruct Schwann cells to dedifferentiate, proliferate, and transform into a repair phenotype (48), thereby facilitating the regeneration of damaged axons and myelin sheaths and ultimately improving proprioceptive function.

Third, the movement characteristics of TC frequently stimulate the proprioceptors in the ankle dorsiflexor and plantarflexor muscles, promoting cortical plasticity and thereby enhancing proprioception. Among the six typical TC steps, four require landing with the ankle near maximal plantarflexion and lifting the foot with the ankle near maximal dorsiflexion (32). These four fundamental steps are repeatedly employed in 19 of the 24-form TC movements, thereby consistently stimulating proprioceptors (49). Furthermore, the brain exhibits “use-dependent plasticity” (50) and the frequent, deliberate practice of TC facilitates such cortical plasticity. Furthermore, another study (51) also showing that active rehabilitation training recruits additional brain regions beyond the primary motor cortex, a recognized marker of cortical plasticity.

The ankle proprioception was significant improved during plantarflexion and dorsiflexion after 8-weeks BW practice. The study by Zhang was consistent with our results, which reported that, at the 12-week mark, significant improvements in ankle proprioception (during both plantarflexion and dorsiflexion) were observed in the BW practitioners relative to pre-practice measurements (24). The improvement in proprioception from BW may operate through mechanisms similar to those of TC. Specifically: BW appears to upregulate BDNF expression, leveraging its neurotrophic functions to support axonal regeneration (52), while also enhancing Schwann cell activity to promote myelin repair (46), collectively contributing to improved proprioception.

### 4.4 Tai Chi (TC) had superior efficacy in enhancing ankle proprioception

After practice, TC practitioners showed greater improvement in ankle proprioception during plantarflexion compared to BW. The findings of the previous study by Zhang aligned with those of the present study, which demonstrated that TC produced greater enhancement in proprioception during plantarflexion than BW in older adults without PN (24). The result may be attributed to certain biomechanical characteristics of TC. The ankle joint, being the weight-bearing joint closest to the ground, constantly adjusts its position during TC practice (11). As mentioned earlier, TC practice involves four fundamental steps (32), and research has shown that prolonged TC training leads to higher activation of ankle dorsiflexors (such as the tibialis anterior and extensor hallucis longus) (31). Both of which provide significant stimulation to intramuscular proprioceptors (49). TC's movements are not defined by standardized angles but emphasize awareness of joint position and direction of motion (24), which includes controlled dorsiflexion, as well as inversion and eversion (swinging the toes inward/outward), even involving dorsiflexion combined with either inversion or eversion even occur during posture transitions and stepping patterns (24) This provides stronger and more comprehensive stimulation to the dorsiflexor muscles and enhances joint position awareness through repetitive deliberate practice. Testing device stretched these frequently activated dorsiflexors during passive plantarflexion. Since proprioception is fundamentally mediated by stretch-sensitive proprioceptors (53), the dorsiflexors in TC practitioners, being more active and thus potentially possessing enhanced receptor sensitivity, were more responsive to this stretch.

Although BW also involves repetitive ankle dorsiflexion, it is a fundamental activity of daily living with a relatively smaller range of ankle motion. Moreover, conscious awareness of joint position and movement is not specifically emphasized during walking (24).

## 5 Strengths and limitations

The main strengths of this study include: First, the use of a randomized controlled trial design, which provides a high level of evidence for causal inference. Second, the application of objective, quantitative sensory-function assessment tools, reducing subjective bias. Third, strict adherence to the intention-to-treat principle, which preserves the benefits of randomization and makes the results more reflective of real-world conditions. This study also has several limitations. First, the relatively small sample size and the 8-week intervention period may have limited statistical power to detect more subtle differences, and the long-term sustainability of the effects remains to be verified. Second, participants were primarily community-dwelling older adults with mild-to-moderate PN; therefore, caution is needed when generalizing the conclusions to individuals with severe neuropathy or hospitalized populations. Finally, although single-blinding (assessor-blind) was implemented, blinding of participants and instructors was not feasible, an inherent limitation in exercise-intervention research.

## 6 Conclusion

This study demonstrated that while both TC and BW significantly improved plantar tactile sensation and ankle proprioception in older adults with PN, TC was more effective than BW at enhancing MH5 and heel position on the sole, as well as proprioception during plantarflexion. Therefore, TC emerges as a valuable exercise intervention for improving sensation and potentially reducing the risk of falls in this population.

Based on the present findings, several important avenues for future research are recommended. First, future studies should employ neuroimaging techniques (e.g., fNIRS, EEG) to elucidate the central neural mechanisms underlying the superior sensory improvements observed with TC compared to brisk walking in people with PN. Second, further research could stratify participants based on the etiology or specific sensory modality deficits of their PN (e.g., large vs. small fiber involvement) to determine if TC's benefits are universal or particularly salient for certain neuropathy subtypes. Third, longitudinal follow-up studies are needed to determine whether the observed improvements in sensory thresholds translate into clinically meaningful reductions in actual fall rates, enhanced gait stability, and improved quality of life over the long term.

## Data Availability

The raw data supporting the conclusions of this article will be made available by the authors, without undue reservation.
